# Reply to ‘Wiggle-match radiocarbon dating of the Taupo eruption’

**DOI:** 10.1038/s41467-019-12491-0

**Published:** 2019-10-11

**Authors:** Richard N. Holdaway, Brendan Duffy, Ben Kennedy

**Affiliations:** 1 Palaecol Research Ltd, P.O. Box 16569, Christchurch, 8042 New Zealand; 2 School of Biological Sciences, University of Canterbury, Private Bag 4800, Christchurch, 8140 New Zealand; 3 School of Earth Sciences, University of Melbourne, Melbourne, 3010 Australia; 4 Department of Geological Sciences, University of Canterbury, Private Bag 4800, Christchurch, 8041 New Zealand

**Keywords:** Biogeochemistry, Environmental chemistry

**Replying to** Alan G. Hogg et al. *Nature Communications* 10.1038/s41467-019-12532-8 (2019)

We appreciate the opportunity to respond to Hogg et al.’s critique^1^ of HDK18^2^. We present responses to the four arguments and additional data analysis presented by Hogg et al. Hogg et al. focus on the wiggle match and Kaipo Bog (KB) dates out of >40 data points. Our paper focused on the whole data set and trends within it. The date series as a whole reveal an undeniable pattern of younging with distance, irrespective of the suggested minor adjustments in the measurements included or excluded (Fig. 1).

**Fig. 1:**
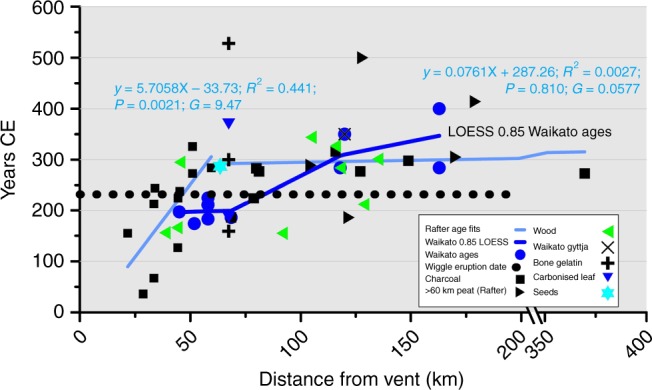
Radiocarbon ages for Taupo eruption are older near the vent. Relationships between radiocarbon ages, distance of sample from vent and relative to tree death for the Taupo First Millennium eruption: median calibrated ^14^C ages for samples against distance from vent system of the Taupo eruption: filled circles, ages measured at the Waikato radiocarbon laboratory, including ages on leaves and seeds from the buried forests at Pureora and Benneydale not included in HDK18; solid dark blue line, fitted LOESS relationship, 0.85 smoothing factor. Other symbols as in legend. Generalised linear model fits for median calibrated dates for revised ^14^C age data set shown by grey lines, equations and statistics, for samples < 60 km and >60 km from the vent. Broken black line, current wiggle-match eruption date

Hogg et al. claim that our ^14^C-date compilation is flawed, with at least 18 additional ages missing and that we include results with large standard errors, and from a range of different studies. We have prepared an updated spreadsheet showing all Taupo radiocarbon dates (apart from the wiggle match and modelled KB date), highlighting those included/excluded in HDK18 (Supplementary Table 1).

Our criteria for inclusion are that the ^14^C date has been published, with stratigraphic control, and no evidence of significant in-built age or reworking, and that it has not been modelled from a date series (e.g., Kaipo Bog). We did not apply an arbitrary cutoff standard error in the date series as the calibrated date distributions take each standard error into consideration. As can be seen in our original Fig. 1^2^ and Supplementary Table 1, the pattern of calibrated date distributions is apparent regardless of the original age standard errors and carbon source(s) and associated methodology and treatment. We used median ages in the analyses because there are equal likelihoods of the actual date being older or younger. Of the 18 missing dates, 8 remain excluded because of uncertainties or lack of information on their stratigraphic relationship to the Taupo tephra, accounting for several of the oldest and youngest dates, as below.

The eight were excluded for the following reasons (also set out in Supplementary Table 1): no published context was found for NZ5531; no location data were found for Wk1502, NZ503 or NZ869; the stratigraphic context was said to be doubtful for Wk424 and NZ525; NZ1060 was from the same site as NZ1059 (included) but reported to below the tephra and much older; NZ160 was reported to be not adjacent to the tephra and to be too young (1300 ± 80 conventional radiocarbon age).

Five further ages on seeds and leaves^3^ were inadvertently omitted from the table, and are now included. The seed and leaf ages provide a two-sigma range for the eruption of 130–320 CE^3,4^, and hence while not resolving the eruption date^4^ they do extend its possible window. The 48 dates are replotted, by laboratory, in Fig. 1. Regardless of whether inclusion criteria are set loosely (including all dates), or stringently (e.g., only data from Waikato laboratory (Fig. 1)), calendar age still declines with distance. We acknowledge a systematic inter-laboratory 40-year offset^1,4^, which appears in the 271 CE mean date for an OxCal4.3 wiggle match on the Sparks et al.^5^ series. However, Waikato laboratory and the Rafter laboratory ^14^C ages data series both show consistent age–distance relationships at distances < 60 km from Lake Taupo. The wiggle match series represented by Wk23140 on the outermost rings is one of the older dates and consistent with the age–distance relationship (Fig. 1).

Hogg et al.^1^ restrict their comments to contesting the vertical movement of dissolved CO_2_ in groundwater. Although we mostly concur with that position, we envisage the movement of CO_2_ as gas as well as dissolved in groundwater, gas migrates easily upwards along faults, through permeable fractured or porous rock, and through soil and groundwater^6,7^. Topography is irrelevant to this process, with many examples of structurally channelled, magmatic CO_2_ found at local low points in elevated sites^8^. Hogg et al. assert that there are no young faults to channel the CO_2_ but the topography clearly shows a scarp and basin along strike of the Pureora site, with uplifted bedrock on the footwall of what is presumably a normal fault. Our view remains that CO_2_ degassed from the basaltic sill system^9^ migrates vertically and along normal faults and through permeable fractured rock to the surface, where it can directly enter the atmosphere beneath a canopy, or first enter groundwater and be redistributed (Fig. 2). The viability of the gaseous CO_2_ to then be incorporated into tree carbon is additionally supported by increasing numbers of studies that report locally sourced CO_2_ as a modifier of the isotopic signature of carbon in trees^10^.

**Fig. 2:**
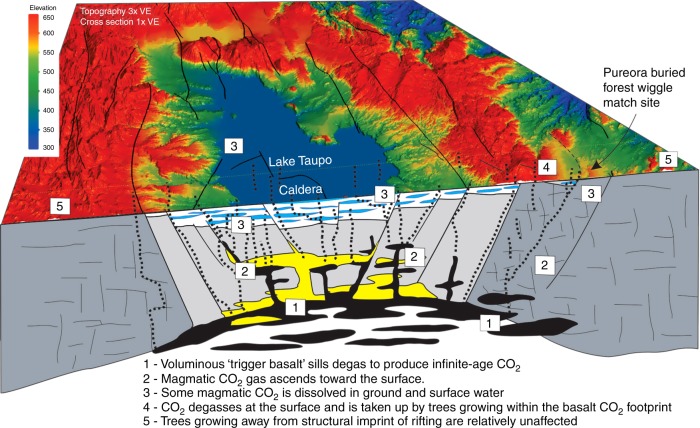
Pathways of magmatic CO_2_ to the surface. Conceptual block-diagram beneath a 25 m Digital Elevation Model (publicly available Alos satellite global data, 1 arc-second resolution), illustrating our proposed mechanism and pathways for CO_2_, from the basaltic sills to the sites of carbon analysis, and highlighting potential fault scarps in the area of the Pureora forest

Hogg et al.^1^ rightly point out that deviation from a straight line does not of itself indicate sampling of a biased atmosphere. We agree that deviation from an appropriately matched SHCal13 wiggle could test our hypothesis of progressive sampling of a ^14^C-diluted atmosphere before an eruption. However, the analysis reported in Hogg et al.’s Table 1^1^ is not appropriate to test our hypothesis. Our hypothesis can only be tested with the ^14^C in the Pureora Tanekaha (*Phyllocladus trichomanoides*) tree matched to the appropriate implied younger portion of the curve. It is not appropriate to test a hypothesis using a portion of the curve that the hypothesis itself rejects. Therefore, any comparison of similitude of the 12 oldest and 13 youngest dates to this portion of the SHCal13 curve is not pertinent.

Similarly, the ^14^C plateaus in the Kauri (*Agathis australis*), and in the Huon pine (*Lagarostrobus franklinii)*, in Hogg et al.’s Fig. 1^1^ are, as expected, consistent with the SHCal13 curve and hence the wiggle-matched Pureora Tanekaha tree. Pointing this out adds nothing beyond the original wiggle match agreement and is not an additional test for our hypothesis. We maintain that this fortuitous match has resulted in the exclusion of the implications of the 47 other data points.

We agree that there are many processes that can affect δ^13^C values. However, we propose that the only process that can produce similar polarity wiggles in both ^14^C and δ^13^C to our knowledge is input of CO_2_ with both lower amounts of ^14^C and higher values of δ^13^C. The only available appropriate data series before three eruptions show these concurrent changes in ^14^C and δ^13^C. We therefore find it likely that such input systematically occurred before each eruption separated by centuries to a millennium, as ^14^C and δ^13^C are controlled by independent processes.

The key points in our discussion of the δ^13^C patterns are that the curve for each tree plateaued before the tree was killed by an eruption, that the timing of each δ^13^C plateau matches that in the SHCal13 ^14^C calibration curve, and that the pattern is repeated in all three trees associated with the two other eruptions.

Bracketing ages for both the Kaharoa and Taupo tephras in the Kapouatai raised bog (KRB)^11^ were measured on peat^12–15^. The KB^12–15^ chronology was also developed on peat dates, but without bracketing ages for either the Taupo or Kaharoa. KRB provides the best stratigraphically constrained sequence away from possible magmatic carbon flux from deeper TVZ basalts.

Peat is a non-preferred dating material, but if the KB peat ages are accepted, the KRB peat ages for the Taupo eruption should be, too, especially as the calibrated date ranges for the Kaharoa ages (Wk1013; Wk1014), in the same measurement series as the Taupo ages, enclose the Kaharoa wiggle match date (Fig. 3a). We suggest that the KRB ages Wk1015 and Wk1016 imply a date c. 350CE (Fig. 3b) for the Taupo eruption.

**Fig. 3:**
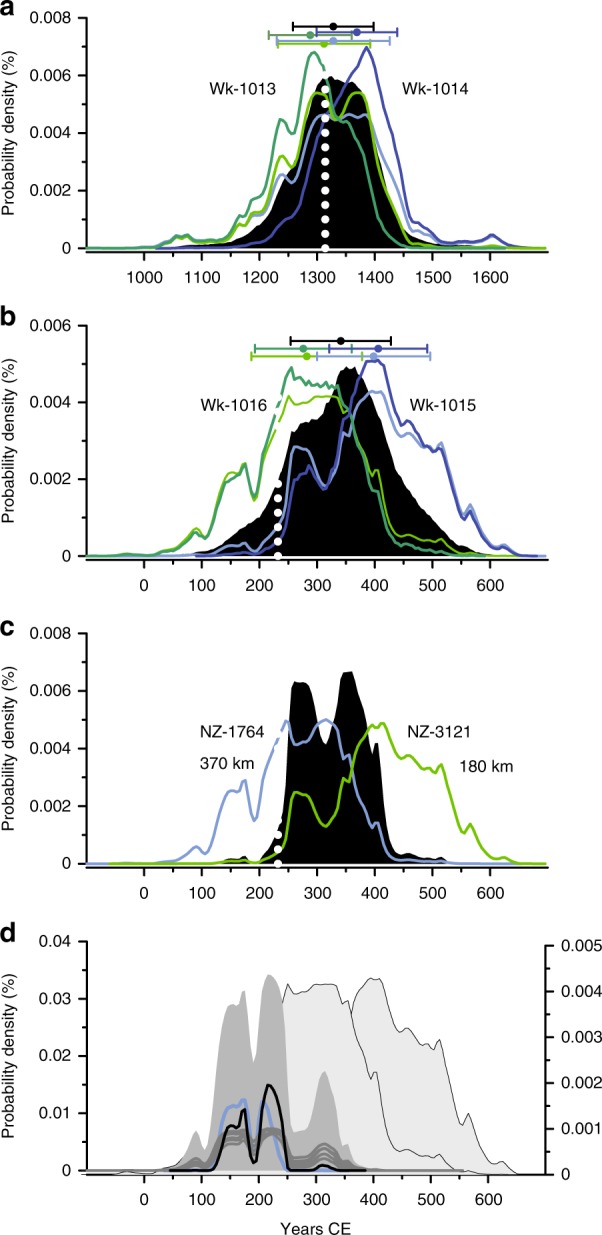
Distant sites yield younger eruption dates. Calibrated ^14^C age distributions for samples bracketing the Kaharoa (Mt Tarawera) and Taupo tephras at sites remoted from the volcanoes and for seeds and leaves from the buried forests at Benneydale and Pureora (near the wiggle match tree). **a** Unmodelled (light green and blue) and modelled (dark green and blue) SHCal13 distributions for Waikato laboratory ^14^C ages (without 40-year offsets attributed to Rafter laboratory ages) on peat samples bracketing the 14th century CE Kaharoa tephra at Kopouatai Bog, 120 km from Mt Tarawera, 160 km from Taupo vent; filled distribution, OxCal4.3.2. transition between the bracketing ages; white dotted line, wiggle match date for Kaharoa eruption; colour-coded means and standard deviations for calibrated distributions above. **b** As for **a**, but for Waikato ^14^C ages, in the same series as the Kaharoa ages, on peat bracketing the Taupo tephra lower in the Kopouatai Bog sequence: white dotted line, wiggle match age for Taupo eruption. **c** SHCal13 distributions for capping sample ^14^C ages the Taupo tephra at two sites distal to Taupo vent (distances below date numbers); calibrated date distribution for NZ1764 (small burnt stumps below tephra) has a second (higher) peak between 300 and 350 CE, and the dominant peak for NZ3121 (decaying vegetation surrounding tephra) approaches 400 CE; filled distribution, OxCal4.3.2 modelled combination of NZ1764 and NZ3121. **d** SHCal13 calibrated date distributions for leaves and seeds from Taupo-killed forests: grey lines, individual ages; black line, OxCal4.3.2 modelled combination of the five ages; dark fill, aligned and summed calibrated date probabilities for the five ages; light filled distributions, calibrated date distributions for Wk1016 and Wk1015 bracketing Taupo tephra at Kopouatai Bog

An at least mid-4th century CE KRB Taupo date is supported also by ages on the tephra in even more distant sites. At Pataua, 370 km northwest of Taupo, the calibrated ^14^C age (NZ1764) on a small charred stump (identified as manuka, *Leptospermum scoparium*) covered by Taupo tephra extends well past 300 CE^16^ (Fig. 3c). The calibrated date distribution for NZ3121 on swamp vegetation enclosing the Taupo tephra at Ngatea, 180 km from the vent, is centred in the late 4th century CE^16^ (Fig. 3c). Results from both laboratories on samples definitively lacking a magmatic CO_2_ source support a younger Taupo eruption date.

The ^14^C ages on seeds and leaves from the buried forest around the wiggle match tree at Pureora and a few kilometres to the west at Benneydale^3^ are as likely to have been subject to magmatic bias as the wiggle match tree(s). Notwithstanding this possibility, the calibrated date ranges include a minor peak after 300 CE, within the range for the peat beneath the Taupo tephra in KRB (Fig. 3d).

Finally, because Hogg et al.^1,4^ assume no contamination, potential fits elsewhere on the SHCal13 curve were not considered. We explored the possibility of fits elsewhere, using the A_comb_ values of fits (as generated by the OxCal4.3 algorithm) for the effect of allowing for progressively greater levels of contamination by infinite age carbon as indicators of statistical support for the wiggle matches, then plotting the years of tree death against the A_comb_ values (Supplementary Fig. 1). The Hogg et al.^4^ and Sparks et al.^5^ wiggle match age series referenced in HDK18 were both examined. The error bars of the original measurements for the Sparks et al.^5^ series were much higher than for Hogg et al. series, resulting in more compliant matches with the SHCal13 curve and generally high A_comb_ values for the fits. There were several instances of A_comb_ values at other parts of the SHCal13 as high as for that for zero contamination, suggesting that, if the Sparks et al.^5^ age series was the only one available, there would be a series of tree dates with similar support.

The low measurement errors for the Hogg et al. ages resulted in more stringent fits with the SHCal13 curve but there was still a fit with A_comb_ > 60% at a date several centuries later than the zero contamination date (Supplementary Fig. 1). The highest A_comb_ value was actually for 0.5% contamination. The effects of contamination were assessed assuming constant levels, which we have proposed is unlikely with the development of the magma bodies. Ramping the contamination in the second half of the age series had no effect in general, but for the spike c. 11.5% contamination, a 10% ramp (11.5–12.6%) from the mid-point in the series yielded an A_comb_ value of 115.4 for the fit (Supplementary Fig. 1). The presence of more than one fit on the SHCal13 curve falsifies the assumption of no contamination. We suggest the heterogeneity and multiple A_comb_ peaks observed with the Sparks et al.^5^, and Hogg et al.^4^ comparison (Supplementary Fig. 1) supports the possibility that contamination varied in time and space.

In summary, following a rigorous reanalysis of the data, we suggest that the pattern in the geographically dispersed corpus of ^14^C ages for the eruption remains indicative of magmatic carbon bias. On the basis of the present data, we conclude that final resolution of this debate and testing of our hypothesis is contingent on obtaining additional ^14^C measurements well beyond the influence of magmatic CO_2_ and/or high-resolution dating of the eruption by methods other than radiocarbon at Taupo and further looking for this effect at other volcanoes.

## Supplementary information

Supplementary Information

## Data Availability

The authors declare that the data supporting the findings of this study are available within the paper and its supplementary information files. Other details pertaining to the data are available from the corresponding author upon reasonable request.
