# Supplementary material for: Reply to ‘Wiggle-match radiocarbon dating of the Taupo eruption’
Source: Nat Commun. 2019 Oct 11;10:4668. doi: 10.1038/s41467-019-12491-0 (PMC6789094; doi:10.1038/s41467-019-12491-0)
Supplement: Supplementary file 1 — Supplementary Information [file 41467_2019_12491_MOESM1_ESM.pdf]

Supplementary Information

Reply to: Wiggle-match radiocarbon dating of the Taupo eruption

Richard N. Holdaway[1,2], Brendan Duffy[3] & Ben Kennedy[4]

Supplementary Figure 1

Supplementary Table 1

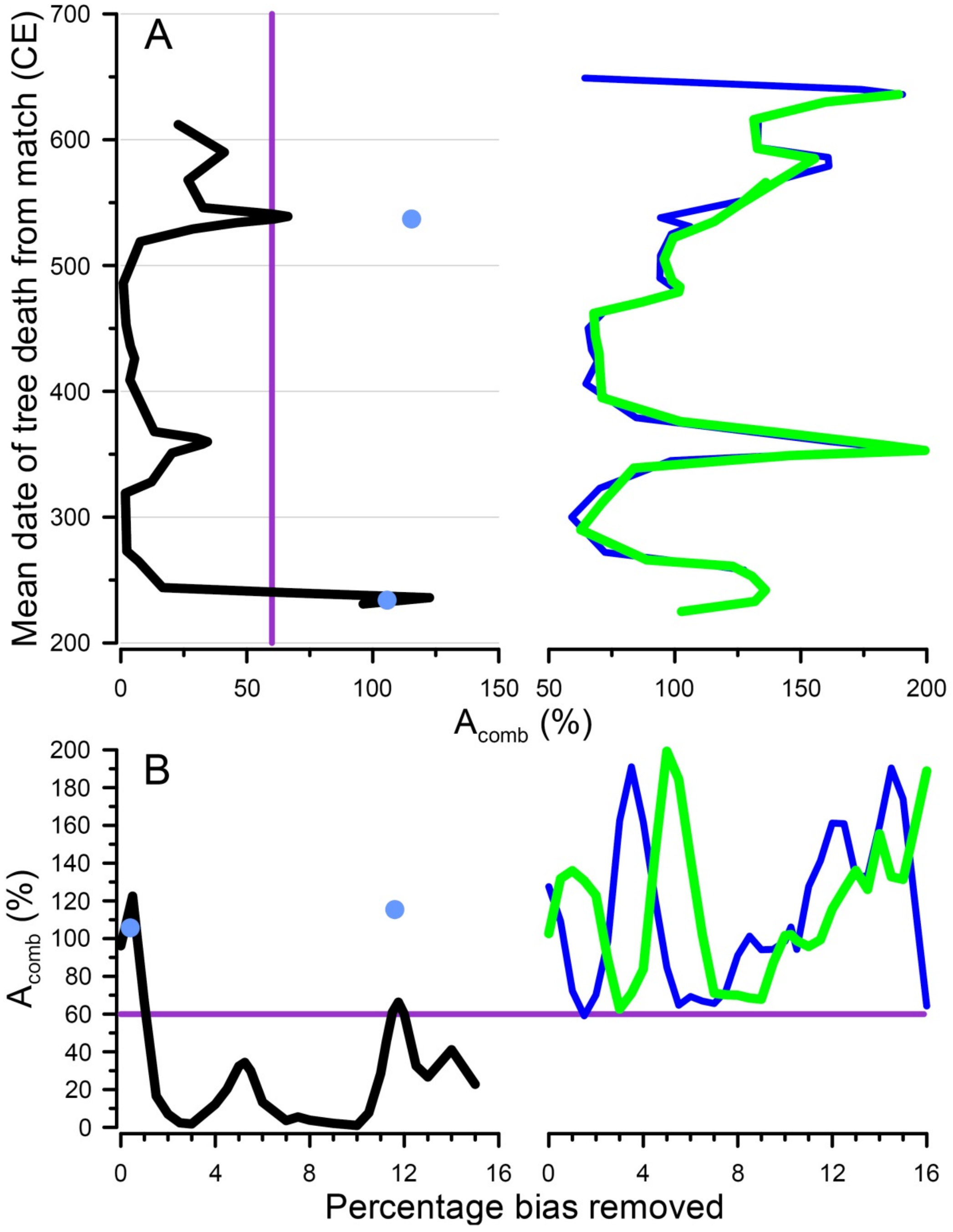

Supplementary Figure 1 Other matches for wiggle date series, with bias removed. Values of A_{comb} for different percentages of contamination removed from wiggle match series conventional radiocarbon ages from (black) Hogg *et al*, (blue) Sparks *et al*. as reported, and (green) Sparks *et al*. laboratory offset removed. **A**, mean dates of tree death for A_{comb} values for increasing percentages of contamination removed. Curves start at 0% contamination as assumed in initial publication of wiggle matches. **B**, A_{comb} values for increasing percentages of contamination removed. Purple, critical value of A_{comb}. Blue symbols, A_{comb} values and tree death date when conventional radiocarbon ages are ramped in second halves of series (as in our model for 0% (to 0.52%) and 11.5% (to 12.6%) contamination, respectively. As with ramping at 0% contamination, ramping of various extents for contamination c. 5% did not generate higher A_{comb} values than peak without ramping: only the peak at c.12% gave a higher A_{comb} value with ramping.

Supplementary Table 1 Radiocarbon data set for date of Taupo eruption. Radiocarbon ages considered for, and used, in analyses, with location relative to Taupo eruption vent system, median calibrated ages (SHCal13 curve), dated material, source references, and comments on reason(s) for exclusion. Colours highlight the ubiquitous distance-age relationship, especially for wood and charcoal. Red = old and close, blue = young and far.

Supplementary Table 1 Radiocarbon data set for date of Taupo eruption. Radiocarbon ages considered for, and used, in analyses, with location relative to Taupo eruption vent system, median calibrated ages (SHCal13 curve), dated material, source references, and comments on reason(s) for exclusion. Colours highlight the ubiquitous distance-age relationship, especially for wood and charcoal. Red = old and close, blue = young and far.

Lab no.	Location relative to vent		CRA	Error	Median age (SHCal13)		Material	Base reference (Entry no.)		Froggatt & Lowe (1990)		Foot note	Comment
	Bearing (°)	Distance (km)			BP	CE				Ref. nos.	Rating		
NZA6636	130.7	67.4	1775	93	1650	300	bone gelatin	Holdaway (1999)			1		
NZA7183	130.7	67.4	1570	68	1422	528	bone gelatin	Holdaway & Beavan (1999)			1		
NZA8559	130.7	67.4			1791	159	bone gelatin	Holdaway & Beavan (1999)			1		
NZA7532	130.7	67.4	1871	67	1580	370	carbonized leaf	Holdaway & Beavan (1999)			1		
NZA8226	130.7	67.4	1715	69	1764	186	carbonized leaf	Holdaway & Beavan (1999)			1		
NZ176	44.6	21.6	1960	70	1795	155	charcoal	Froggatt & Lowe (1990)	22	14,8	1		
NZ82	22.03	28.6	2040	50	1914	36	charred wood	Froggatt & Lowe (1990)	7	14,8	2		
NZ3	237.4	33.5	1970	150	1883	67	charcoal	Froggatt & Lowe (1990)	3	14,8	1		
NZ175	32.5	33.5	1850	100	1737	213	charcoal	Froggatt & Lowe (1990)	21	14,8	1		
NZ1	132.32	34	1820	150	1706	244	charcoal	Froggatt & Lowe (1990)	2	14,8	1		
NZ4	204.3	44.3	1920	150	1823	127	charcoal	Froggatt & Lowe (1990)	4	14	2		
NZ163	14	44.4	1840	50	1726	224	charcoal	Froggatt & Lowe (1990)	13	14,8	1		
NZ162	353.4	45.2	1830	70	1712	238	charcoal	Froggatt & Lowe (1990)	12	14,8	1		
NZ173	7.3	51	1750	50	1624	326	charcoal	Froggatt & Lowe (1990)	19	14,8	1		
NZ174	7.3	51	1800	100	1677	273	charcoal	Froggatt & Lowe (1990)	20	14,8	1		
NZ170	231.9	59.5	1800	50	1666	284	charcoal	Froggatt & Lowe (1990)	17	14,8	1		
**WK928**	16.2	68.5	1870	60	1764	186	charred wood	Froggatt & Lowe (1990)	41	f	1		
NZ1548	42.5	78.7	1840	50	1726	224	charcoal	Froggatt & Lowe (1990)	31	56,7,21	1		
NZ172	37.5	79.9	1800	100	1667	283	charcoal	Froggatt & Lowe (1990)	18	14,8	1		
NZ183	111.3	81.1	1840	70	1673	277	charcoal	Froggatt & Lowe (1990)	23	14,8	1		
NZ158	141.5	115.8	1760	80	1634	316	charcoal	Froggatt & Lowe (1990)	8	14,8	1		
NZ38	335.1	127.1	1800	70	1673	277	charcoal	Froggatt & Lowe (1990)	6	14,8	1		
NZ37	332.1	148.9	1780	60	1652	298	charcoal	Froggatt & Lowe (1990)	5	14,8	1		
NZ1764	339.8	369.6	1805	65	1677	273	charred wood	Pullar *et al.* (1977)		-	-		
NZ5531			1890	70			charcoal	Froggatt & Lowe (1990)	33	z	2	d	Not published anywhere for its context to be properly evaluated.
**WK215**	**332.4**	**120**	**1730**	**60**	**1600**	**350**	**gyttja**	**Froggatt & Lowe (1990)**	**39**	**16,25,28,12**	**2**		
NZ5610A	131.2	104.6	1790	65	1661	289	peat/wood	Froggatt & Lowe (1990)	34	20	2		
NZ5610B	*131.2*	*104.6*			*1731*	*219*	*peat/wood*				*4*	*c*	*Recalculation*

NZ1059	42.5	122	1870	60	1764	186	peat	Froggatt & Lowe (1990)	29	37, 39, g	1		
NZ7013	331.5	128.2	1600	55	1450	500	peat	Froggatt & Lowe (1990)	36	c	2	a	
**WK1015**	**348.3**	**162.8**	**1690**	**80**	**1550**	**400**	**peat**	**Froggatt & Lowe (1990)**	**42**	**d**	**1**	**b**	
**WK1016**	**348.3**	**162.8**	**1790**	**80**	**1664**	**286**	**peat**	**Froggatt & Lowe (1990)**	**43**	**d**	**1**	**b**	
NZ502	80.9	170.3	1770	70	1645	305	peat	Froggatt & Lowe (1990)	24	9,39	1		
NZ3121	347.2	179.2	1680	70	1536	414	peat	Froggatt & Lowe (1990)	32	55	3		
**WK1502**			**1890**	**50**			**peat**	**Froggatt & Lowe (1990)**	**1**	**l**	**2**	**d**	**No location data**
NZ503			1900	60			peat	Froggatt & Lowe (1990)	25	9, 39	2	d	**No location data**
NZ1060			2090	60			peat	Froggatt & Lowe (1990)	30	37, 39, g	2	d	**Same site as NZ1059 but below tephra and much older**
NZ7442	306.6	63.5	1795	55	1663	287	seeds	Froggatt & Lowe (1990)	37	g	1	a	
NZ168	38.7	38.5	1980	40	1794	156	wood	Froggatt & Lowe (1990)	16	14,8,43	1		
NZ164	12.3	43.9	1890	70	1784	166	wood	Froggatt & Lowe (1990)	14	14,8	1		
NZ161	353.4	45.2	1780	80	1655	295	wood	Froggatt & Lowe (1990)	11	14,8	1	a	
**WK23140**	**321**	**51.8**	**1874**	**18**	**1827**	**123**	**wood cellulose**	**Hogg *et al.* (2011)**					
NZ165	342.9	91.5	1900	70	1795	155	wood	Froggatt & Lowe (1990)	15	14,8	1		
NZ5611A	131.2	104.6	1735	65	1606	344	wood	Froggatt & Lowe (1990)	35	20	2		
NZ5611B	*131.2*	*104.6*			*1657*	*293*	*wood*				*4*	*c*	*Recalculation*
NZ5611C	*131.2*	*104.6*			*1587*	*363*	*wood*				*4*	*c*	*Recalculation*
NZ159	141	115.8	1750	80	1623	327	wood	Froggatt & Lowe (1990)	9	14,8	1		
**WK1094**	**39.7**	**118**	**1800**	**50**	**1666**	**284**	**wood**	**Froggatt & Lowe (1990)**	**44**	**j, 62**	**1**	**b**	
NZ7482	34.4	128.6	1850	60	1738	212	wood	Froggatt & Lowe (1990)	38	59	1	a	
NZ524	80.9	135	1775	75	1649	301	wood	Froggatt & Lowe (1990)	26	9,39	1		
NZ160			1300	80			wood	Froggatt & Lowe (1990)	10	8	3	d	Not adjacent to tephra, too young
NZ869			1995	60			wood	Froggatt & Lowe (1990)	28	39	2	d	No location data
**WK424**			**2040**	**50**			**wood**	**Froggatt & Lowe (1990)**	**40**	**16, 26**	**3**	**d**	Doubtful stratigraphy
NZ525			2101	75			wood	Froggatt & Lowe (1990)	27	9, 39	2	d	Doubtful stratigraphy

a	Used but not tabled in HDK18
b	Declared not used in HDK18
c	Duplicate - different 1/2-life
d	Discarded without mention in HDK18

Waikato dates bold

The remainder were either recalculations or declared in the footnote of the original table. The revised table includes 47 ages measured by the Rafter laboratory and 12 by the Waikato laboratory: 10 from Rafter and 1 from Waikato have been excluded, for reasons noted.

We do not cite dates from KB because the Taupo tephra has never been directly dated there. The unit encompassing the Kaharoa and Taupo tephras was truncated by erosion[5].

We have not found published KB dates younger 3000 BP [3,5-8].

References

Froggatt, P. C. & Lowe, D. J. A review of late Quaternary silicic and some other tephra formations from New Zealand: Their stratigraphy, nomenclature, distribution, volume, and age. *N. Z. J. Geol. Geophys.* **33**, 89-109 (1990).

References in Froggatt & Lowe (1990) are listed in HDK18, Supplementary material.

Hogg, A., Lowe, D. J., Palmer, J., Boswijk, G. & Ramsey, C. B. Revised calendar date for the Taupo eruption derived by ^{14}C wiggle-matching using a New Zealand kauri ^{14}C calibration data set. *The Holocene* **22**, 439-449.

Holdaway, R. & Beavan, N. Reliable ^{14}C AMS dates on bird and Pacific rat *Rattus exulans* bone gelatin, from a $CaCO_3$-rich deposit. *J. R. Soc. N. Z.* **29**, 185-211 (1999).

Pullar, W.A.; Kohn, B.P.; Cox, J.E. 1977. Airfall Kaharoa Ash and Taupo Pumice, and sea-rafted Loisels Pumice, Taupo Pumice, and Leigh Pumice in northern and eastern parts of the North Island, New Zealand. *N. Z. J. Geol. Geophys.* **20**, 697-717.
